# Primary Retroperitoneal Mucinous Cystadenocarcinoma: A Case Report

**DOI:** 10.7759/cureus.39983

**Published:** 2023-06-05

**Authors:** Saroja Devi Geetha, Louis Kavoussi, Rebecca Thomas, Deepika Savant

**Affiliations:** 1 Pathology, Northwell Health, Greenvale, USA; 2 Urology, Northwell Health, Greenvale, USA

**Keywords:** malignancy, cystadenocarcinoma, mucinous neoplasms, retroperitoneal mass, retroperitoneal cyst

## Abstract

Mucinous neoplasms are commonly seen in the ovaries and pancreas. Their occurrence in the retroperitoneum is uncommon. We present a case of a retroperitoneal mucinous cystadenocarcinoma in a 54-year-old female who presented with right flank pain. Imaging demonstrated an 8.6 × 7.9 cm mass at the anterior surface of the lower pole of the right kidney, suspicious for renal cell carcinoma. Serum tumor markers carbohydrate antigen 19-9 (CA 19-9) and cancer embryonic antigen (CEA) were within normal limits, and cancer antigen 125 (CA 125) was elevated. Surgical resection of the mass was performed. Intraoperatively, the mass was noted to lie in the retroperitoneum, unattached to the kidney. On gross examination, a 10.0 × 7.0 × 7.0 cm unilocular cystic structure with red-brown mucoid material was present. The inner lining was mostly smooth with areas of excrescences, covering less than 5% of the surface area. Microscopic examination showed cystic areas lined by mucinous epithelium with an underlying ovarian-type stroma. Solid areas showed features of a borderline papillary mucinous tumor with invasive carcinoma. A diagnosis of mucinous cystadenocarcinoma was made. Their occurrence in the retroperitoneum is unusual. Although rare, this entity should always be considered in the differential diagnosis of retroperitoneal cystic lesions.

## Introduction

Mucinous cystic neoplasms are known to occur in the ovaries and pancreas. However, in rare instances, they are found to occur as primary retroperitoneal masses [1]. They are described more commonly in women than in men. Their presentation is often delayed due to the asymptomatic nature of the lesion, especially when small. Histologically, they can be classified as benign, borderline, and malignant, with benign lesions being the most common type. Malignant lesions with invasion are rare; we present one such case.

## Case presentation

A 54-year-old female presented with complaints of right flank pain. The pain had been progressively increasing in intensity over the previous year. Her past medical and surgical history was significant for hypothyroidism, hyperlipidemia, depression, and sleeve gastrectomy for morbid obesity, with hiatal hernia repair. Physical examination was within normal limits.

**Figure 1:**
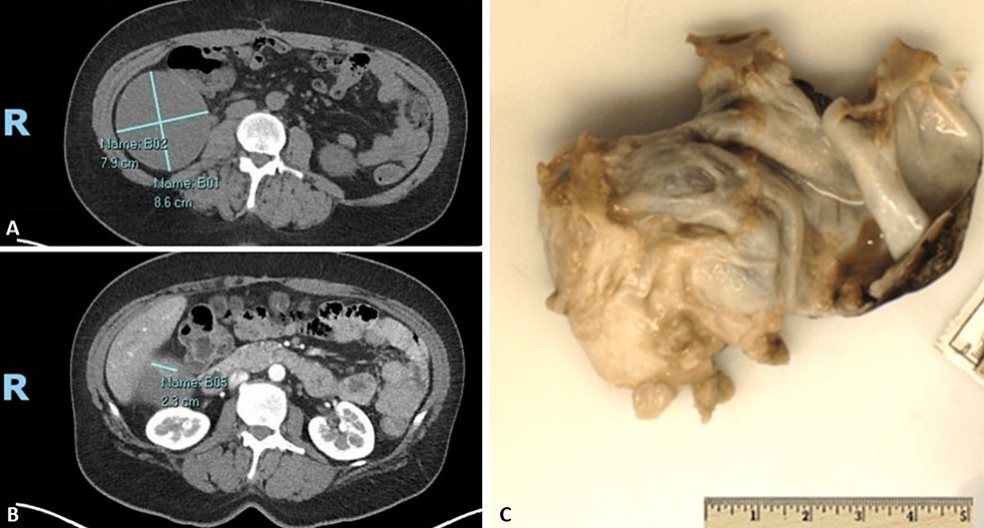
CT of the Abdomen and Gross Findings A: CT of the abdomen with contrast demonstrating an 8.6 × 7.9 cm mass at the anterior lower pole of the right kidney. B: CT of the abdomen with contrast demonstrating the largest mural nodule within the mass measuring 2.3 cm. C: Gross appearance of the unilocular cyst after evacuation of cyst contents. CT: computed tomography

Multiple modalities of imaging were obtained. CT with contrast demonstrated an 8.6 × 7.9 cm mass at the anterior surface of the lower pole of the right kidney with peripherally enhancing mural nodules at the superior and inferior margins of the mass (Figure 1A). The largest nodule (2.3 cm) was at the superior aspect of the mass (Figure 1B). These findings were consistent with a Bosniak IV cystic renal mass and were considered highly suspicious for renal cell carcinoma. A comparison CT from seven years earlier demonstrated that this lesion had been present and classified as Bosniak type II F.

Serum tumor markers carbohydrate antigen 19-9 (CA 19-9) and cancer embryonic antigen (CEA) were within normal limits; however, cancer antigen 125 (CA 125) was elevated at 46 U/mL (normal value: 0-35 U/mL). Given these findings, a decision was made to remove the mass laparoscopically as a partial right nephrectomy. Intraoperatively, a large mass was seen protruding from the retroperitoneum, just lateral to the colon and adherent to it. It was not attached to the kidney. Intraoperative ultrasound of this mass demonstrated a homogenous structure. The mass was then carefully removed and sent for pathological examination.

A gross examination of the mass revealed a 10.0 × 7.0 × 7.0 cm unilocular cystic structure with a smooth surface and attached adipose tissue (Figure 1C). The cyst was filled with red-brown mucoid material. The inner lining of the cyst was smooth and tan-pink with excrescences ranging from 0.2 to 1.2 cm in greatest dimension and covering less than 5% of the cyst lining.

**Figure 2:**
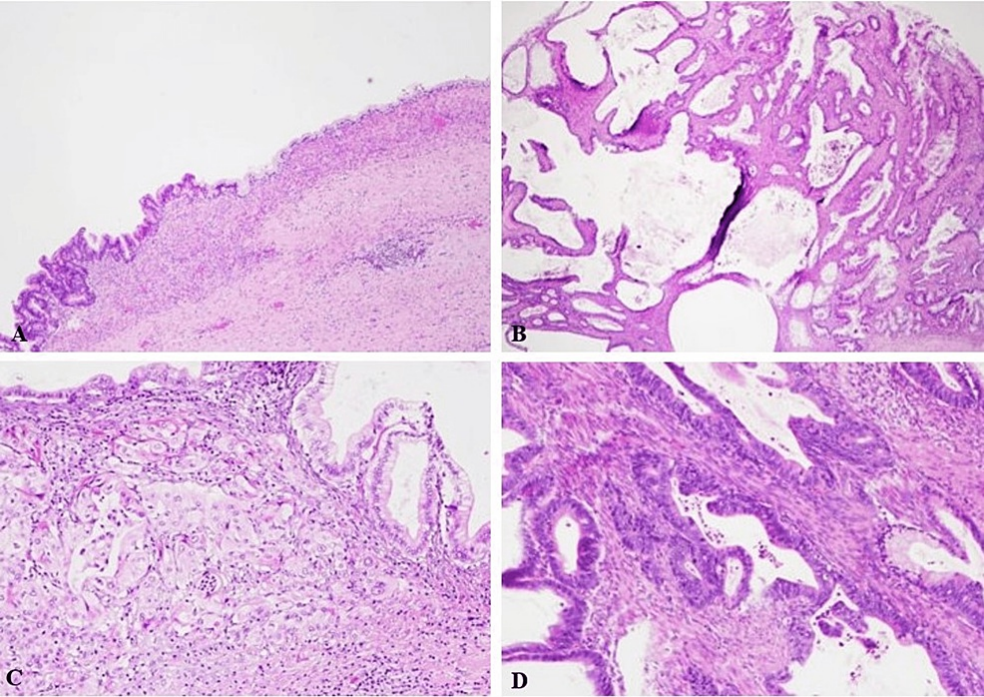
Microscopic Images of the Lesion A: Cyst lining composed of mucinous epithelium (H&E, 4×). B: Solid area of the tumor lined by mucinous epithelium exhibiting borderline papillary features (H&E, 2×). C: Solid areas composed of cells with features of invasive carcinoma (H&E, 10×). D: Cystic areas lined by mucinous epithelium with underlying ovarian-type stroma (H&E, 10×).

**Figure 3:**
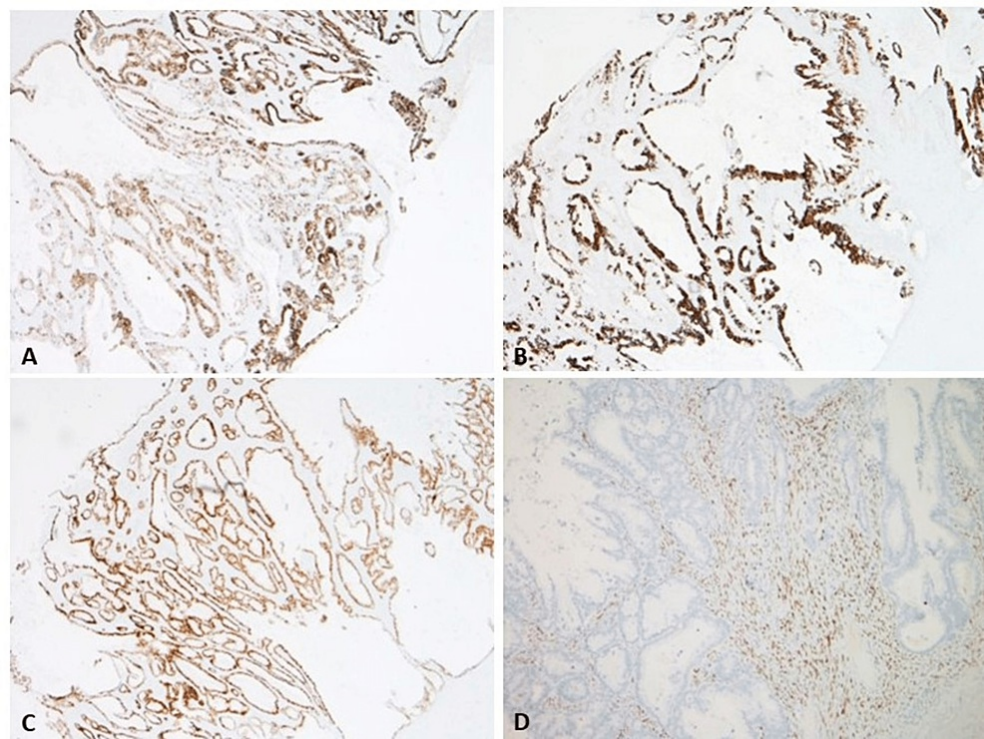
Immunostains A: Tumor cell positivity with CK7 (2×). B: Tumor cell positivity with CK20 (2×). C: Tumor cells showing diffuse positivity with CDX2 (2×). D: Stromal cells positive for ER (2×). CK7: cytokeratin 7, CK20: cytokeratin 20, CDX2: caudal-type homeobox transcription factor 2, ER: estrogen receptor

Histopathologic examination showed that the tumor was found to be partly cystic and partly solid (Figure 2B). The cystic areas were lined by mucinous epithelium with an underlying ovarian-type stroma (Figure 2D). The solid areas showed features of a borderline papillary mucinous tumor with areas of moderately differentiated invasive adenocarcinoma with mucinous features (Figure 2C). No renal parenchyma was identified. Immunohistochemical studies were performed. The ovarian-type stroma was positive for estrogen receptor (ER) (Figure 3D) and negative for cluster of differentiation 10 (CD10). The epithelial cells were strongly positive for cytokeratin 7 (CK7), caudal-type homeobox transcription factor 2 (CDX2), and cytokeratin 20 (CK20) (Figure 3A-3C) and focally positive for paired-box gene 8 (PAX8) and special AT-rich sequence-binding protein 2 (SATB2). Given these findings, a diagnosis of primary retroperitoneal mucinous cystadenocarcinoma was made.

## Discussion

Ovarian and pancreatic mucinous cystic neoplasms are very well known. However, primary retroperitoneal mucinous neoplasm is rare. It was first described in 1924 by Handfield‐Jones [1]. The rarity of this lesion in this region can be attributed to the absence of epithelial cells in the retroperitoneum. Many theories have been proposed for the occurrence of this tumor in the retroperitoneum [2]. One theory is the occurrence of ectopic ovarian tissue in the retroperitoneum. This theory supports the presence of ovarian stroma and its predominant occurrence in females. However, no ovarian follicles have been identified in the cyst wall, and occasionally, these lesions tend to occur in men [3,4]. The second theory is the presence of multipotent mesothelial cells in the retroperitoneum, which undergo mucinous metaplasia, giving rise to these lesions [5]; however, the presence of ovarian stroma cannot be explained.

As noted, these tumors mostly occur in women, although few cases have been reported in men [6]. In most cases, patients are asymptomatic, occasionally presenting with abdominal pain. Histologically, mucinous neoplasms are classified into benign, borderline, and malignant, just like their ovarian counterparts [7,8]. Benign mucinous cystadenoma is the most common subtype and has a very good prognosis. Borderline mucinous cystadenoma shows papillary projections and is less common. Malignant tumors are rare, show invasion, and have the worst prognosis.

The correct diagnosis prior to surgery is challenging. Although CA 125 was elevated in our patient, serum marker assays are not usually useful, as their expression is variable. Additionally, in our patient, the cyst appeared to have arisen from the kidney radiologically with a strong suspicion of renal cell carcinoma. However, intraoperatively, it was seen to be separate from the kidney, and microscopic examination confirmed the absence of renal tissue. Thus, diagnosis is almost entirely based on histopathology.

Usually, these lesions tend to begin as benign tumors and proceed to acquire malignant features. This theory holds in our case as the patient had a radiologically evident lesion a few years prior that was smaller in size and progressed to the current lesion. Histologically, the presence of borderline areas and foci of invasion support the transformation from a benign cystadenoma to a malignant cystadenocarcinoma.

Complete surgical resection of the retroperitoneal mass is recommended because of the possibility of malignancy; complications such as infections are also likely. Although a few authors suggest prophylactic bilateral salpingo-oophorectomy, with or without hysterectomy, to improve survival, there are no studies to support this recommendation [9-11]. In cases that have been reported so far, one patient presented with bone metastases [12], and in another case, there were widespread metastases, which were diagnosed only at autopsy [13]. The metastases in these two cases followed complete surgical resection. Therefore, chemotherapy is advised if associated with invasion or intraoperative spillage to reduce the risk of metastasis [14]. The presence of solid areas, mural nodules, and sarcoma-like or anaplastic components have been found to be associated with the aggressive behavior of this tumor [15]. Also, males are at higher risk of developing malignant lesions as all cases of retroperitoneal neoplasms described in males had a malignant focus [16-19]. Postsurgical follow-up with serum markers if elevated prior to surgery can help monitor for recurrences. Our patient is currently 11 months post this diagnosis and is on active surveillance.

## Conclusions

Mucinous cystic neoplasms are rare primary retroperitoneal masses, more commonly found in women, and are typically asymptomatic, leading to a delayed diagnosis. Histopathologic examination plays a key diagnostic role in these cases as they are clinically and radiographically challenging, often being diagnosed as ovarian or renal masses.
